# Demystifying the mechanism of action of professional facial peeling: In‐vivo visualization and quantification of changes in inflammation, melanin and collagen using Vivascope® and ConfoScan®

**DOI:** 10.1111/dth.15846

**Published:** 2022-10-03

**Authors:** Shazli Razi, Vinay Bhardwaj, Samantha Ouellette, Samavia Khan, Chloe Azadegan, Thomas Boyd, Babar Rao

**Affiliations:** ^1^ Department of Dermatology and Pathology Rao Dermatology Atlantic Highlands New Jersey USA; ^2^ Department of Global Personal Care and Skin Health R&D Colgate‐Palmolive Piscataway New Jersey USA; ^3^ Robert Wood Johnson Medical Center Rutgers University New Brunswick New Jersey USA; ^4^ School of Arts and Sciences Cell Biology and Neuroscience New Brunswick New Jersey USA

**Keywords:** collagen remodeling, exfoliation, melanin redistribution, peeling, professional peel, reflectance confocal microscopy, Vivascope

## Abstract

Professional peeling using chemicals (chemical peeling) is a popular non‐surgical procedure commonly used for the treatment for photoaging, pigmentary disorders, scarring, fine lines, and wrinkles. The objective of our case study was to elucidate the mechanism of action of professional peels/peeling. For proof‐of‐concept, we used a commercial blended peel containing trichloroacetic acid and lactic acid. The facial peeling was performed by a physician on four subjects. These subjects were followed over time in the clinic to take clinical pictures and monitor surface and anatomical changes in inflammation, melanin, and collagen at regular intervals post‐peel (5 min, 48 h, and day 9). Dermoscope and Vivascope**®** were used to image surface and subsurface anatomical changes, respectively, and ConfoScan**®** was used to quantify aforementioned anatomical changes. Based on Vivascope and ConfoScan analysis, we could see clear visual clinical evidence of controlled injury‐healing mechanism of peel's action: immediate but transient onset of inflammation within 5 min (indicate injury response by skin), followed by melanin redistribution evident at 48 h (indicate activation of skin's defense system), and remodeled fibrous collagen network without any inflammatory cells on day 9 (healing response). To our knowledge, this is the first ever clinical study to deconvolute the mysterious mechanism of action of peels, in‐vivo.

## 1 INTRODUCTION

According to the Aesthetic Plastic Surgery National Databank (https://www.surgery.org/media/statistics), for the past five consecutive years (2015–2020), chemical peeling has been among the most popular non‐surgical procedures in the United States. The modus operandi of chemical peels involves using acids (chemical ablative agents) to peel damaged or dead cells from the dull looking skin surface, a process called chemoexfoliation. This exfoliating effect stimulates growth of epidermis, collagen remodeling, and more evenly distributed melanin.^1^ After a chemical peel, exfoliation may vary depending upon myriad factors such as active ingredients, concentration, and layers of chemical peel applied. Chemical peels cause controlled chemical injury of a specific skin depth (epidermis and/or dermis) with the goal of improving skin texture, firmness, and even tone.^1^, ^2^ This controlled injury, followed by skin regeneration, remodeling and wound healing process may be responsible for improvement in skin texture, even tone, and firmness or anti‐aging effects.^3^

Superficial chemical peels (<30%–35% trichloroacetic acid, TCA) are often combined with other peeling agents such as 10%–70% hydroxy acids (alpha and/or beta) and can typically penetrate up to the superficial papillary dermis. Deep chemical peels can penetrate up to mid reticular dermis and often contain TCA or phenol at a higher concentration.^4^ Lactic acid (20%) and TCA (10%) are two primary active ingredients in the commercial blended peel we used for our investigational research. Lactic acid causes exfoliation by breaking desmosomal bonds between keratinocytes.^5^ TCA has keratocoagulant action and is therefore considered a gold standard peeling agent by some dermatologists. These two active ingredients work synergistically when used together, and because of this synergy, a lower dose of TCA is needed.^4^

There are some studies showing the effect of chemical peeling on melanin, collagen, elastin, epidermal and dermis thickness.^4^ However, these studies either used human skin explants for the chemical peel treatment^4^ or used the classical invasive punch biopsy and histological staining approach to observe changes.^6^, ^7^ One study has demonstrated use of Vivascope to clinically capture the reduction in melanin after peeling.^8^ However, it was not certain that reduction in melanin was due to chemical peel or the daily use of skin brightener after 1 week of peeling. Vivascope images were acquired only before and after peel (no kinetics), which failed to provide any insights on mechanism of chemical peel's action. Further, the previous study investigated 3% retinol peel, which has a different mode of action than commonly used TCA‐based peel. A study on kinetics of changes after peeling has been reported, however, it was done on mice model.^9^ To our knowledge, this is the first ever‐clinical study that aims to investigate the mechanism of chemical peel's action by using non‐invasive optical biopsy imaging (Vivascope 1500) and image analysis (ConfoScan) to monitor markers or indicators of injury and healing in skin.

## 2 METHODS

**CHART 1:**
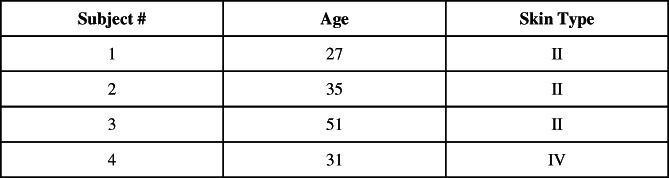
Design of experiment. Subjects were imaged using Vivascope 1500 pre‐peel, and post‐peel (5 min, 48 h, and 9 days) to understand pathophysiology (mechanism of action)

We followed the manufacturer's guidelines for peeling and post‐peeling care. We used a cleanser to thoroughly clean the face followed by pat drying. This was followed by application of a toner via a cotton pad then allowing the skin to dry. Ultra Peel® by PCA SKIN® was first applied on thicker areas of the face (cheeks, forehead, chin, nose) followed by thinner areas (around the mouth) in even strokes. Subjects were provided with post‐procedure care kits (sunscreen, cleanser, moisturizer, and skin procedure ointment). Unlike other peel clinical studies^7^, ^8^, ^10^ there was a 14 days washout period before peeling, and subjects were restricted to use only the supplied post‐peel care products to make sure the changes are due to peeling and not pre‐ or post‐peel care products that can increase peeling efficacy against pigmentation and collagen. Facial peeling was done on four subjects (Chart 1) and confocal microscopy images were obtained post‐peel after 5 min, 48 h, and day 9 using FDA‐approved skin imaging instrument, Vivascope 1500. By obtaining images at regular intervals post‐peel, we are aiming to visualize controlled chemical injury and healing processes to better understand the mechanism of action of chemical peel. Subjects were instructed to take pictures of their face for 5–7 days or until the peeling/exfoliation was finished. Ethical approval statement: Subjects have given their written informed consent to publish their case (including publication of images). The study protocol was reviewed and approved by Advarra IRB.

**FIGURE 1:**
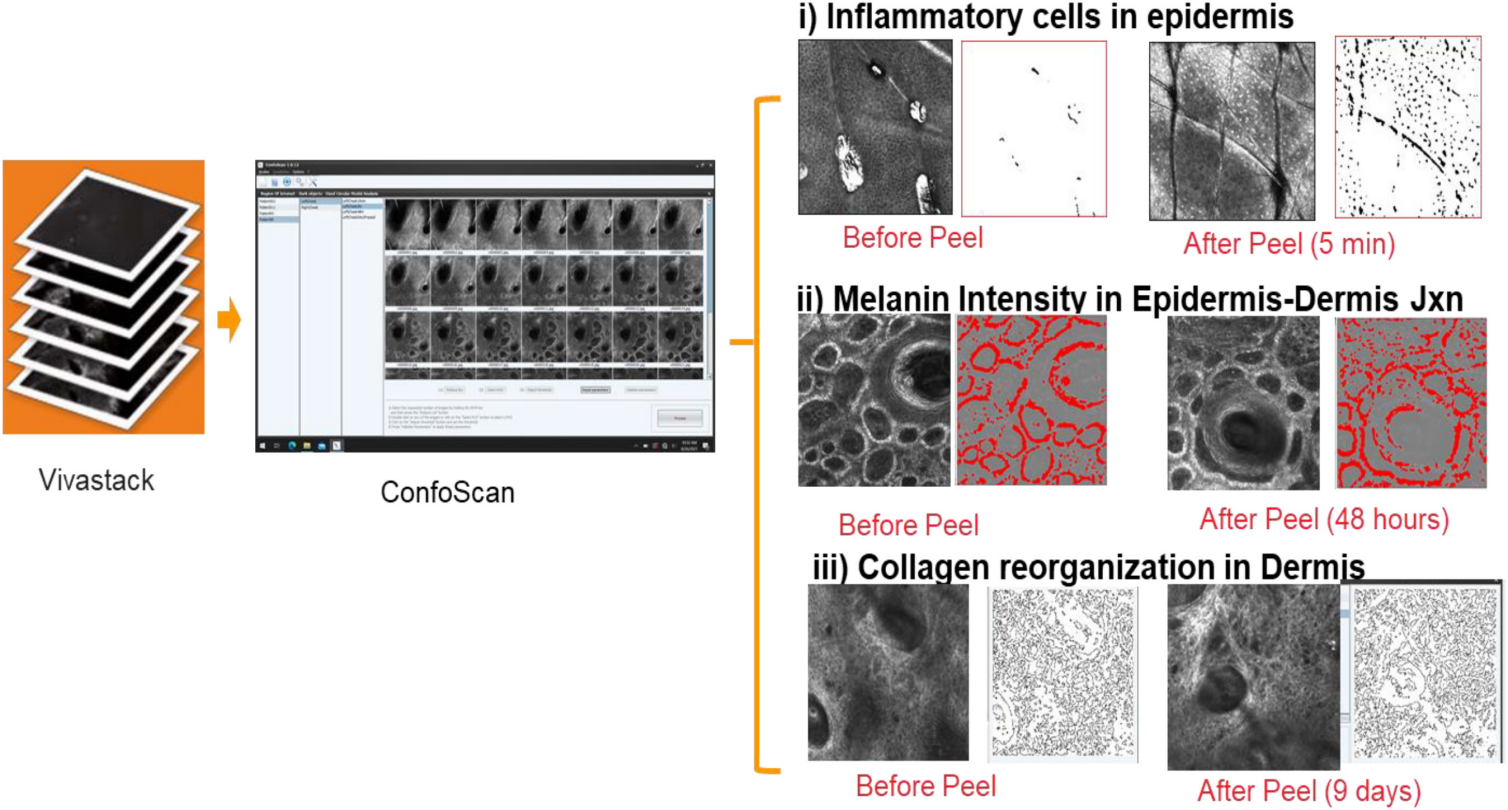
Illustration of image acquisition (Vivascope 1500) and processing (ConfoScan) to visualize and quantify changes in response to chemical peeling

Reflectance Confocal Microscopy images acquired using Vivascope 1500 were analyzed with the help of ConfoScan software version 1.0.12 from Orion technolab, France. Figure 1 illustrates the workflow of the image acquisition and analysis. The ConfoScan software package is exclusively designed and validated for confocal Vivascope images. This software was used to process confocal Vivascope images using algorithms designed to discriminate and quantify black and white structures in Vivascope confocal images based on the shape, size and contrast of those anatomical structures. At least three subsequent images (sections/stacks) were processed to get quantitative information on inflammatory cells (epidermis), melanin (dermal‐epidermal junction) and collagen (dermis). By using both, original and processed images, we aim to visually highlight and quantify changes in inflammation, melanin and collagen.

## 3 RESULTS

### 3.1 Confocal microscopy

#### 3.1.1 Inflammation

**FIGURE 2:**
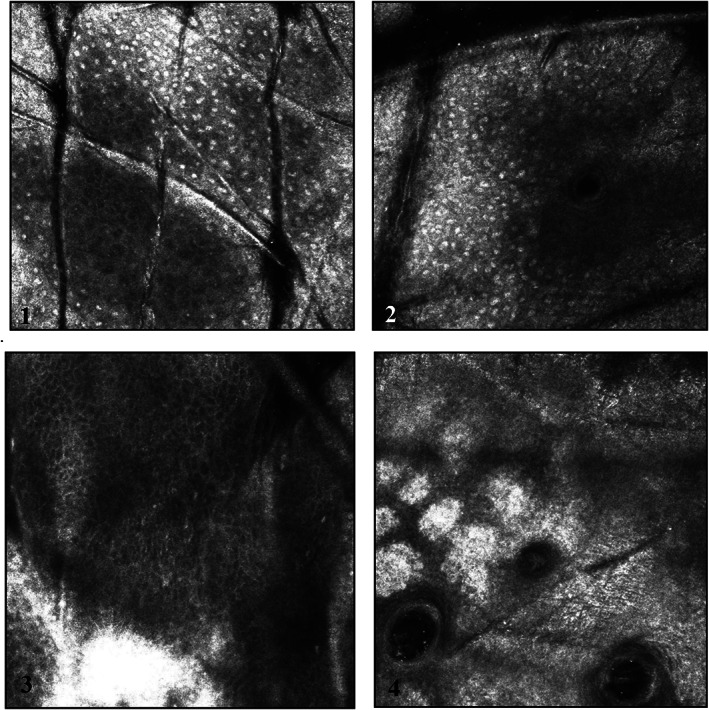
Pattern of inflammatory cells. Pin point inflammatory cells with little melanin redistribution in subject 1 and 2 (images 1 and 2). Globular inflammatory cells with high levels of melanin redistribution and fluid, giving sponge‐like appearance in subject 3 and 4 (images 3 and 4)

**CHART 2:**
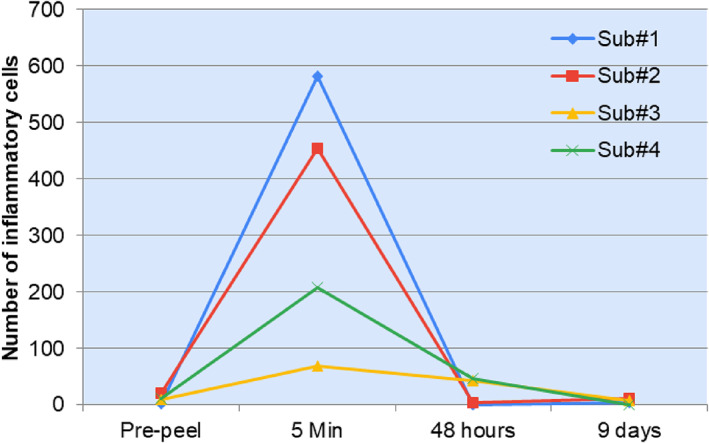
Quantification of inflammatory cells pre‐and post‐peel (5 min, 48 h, and 9 days) to understand inflammation kinetics in response to chemical peel

Inflammatory cells appear as small, bright round or stellar shaped structures without visible nuclei under RCM.^11^ In all four subjects there were clear visual signs of instant but transient inflammation after chemical peeling (Figure 2). In subject number 1 and 2, inflammatory cells were expressed uniformly and had clear pinpoint arrangement with peak inflammation at 5 min, which returned to baseline at 48 h (Chart 2). Both these subjects had similar skin type (Fitzpatrick II, and uniform skin tone) and only one layer of chemical peel was applied. Whereas in subject 3 and 4, inflammation persisted till 48 h and inflammatory cells had spongiosis‐like arrangement (colocalization of inflammatory cells and melanin containing keratinocytes drained with fluid). Subjects 3 and 4 had high melanin content (dark skin type or dark patches on light skin) as compared to subjects 1 and 2.

**FIGURE 3:**
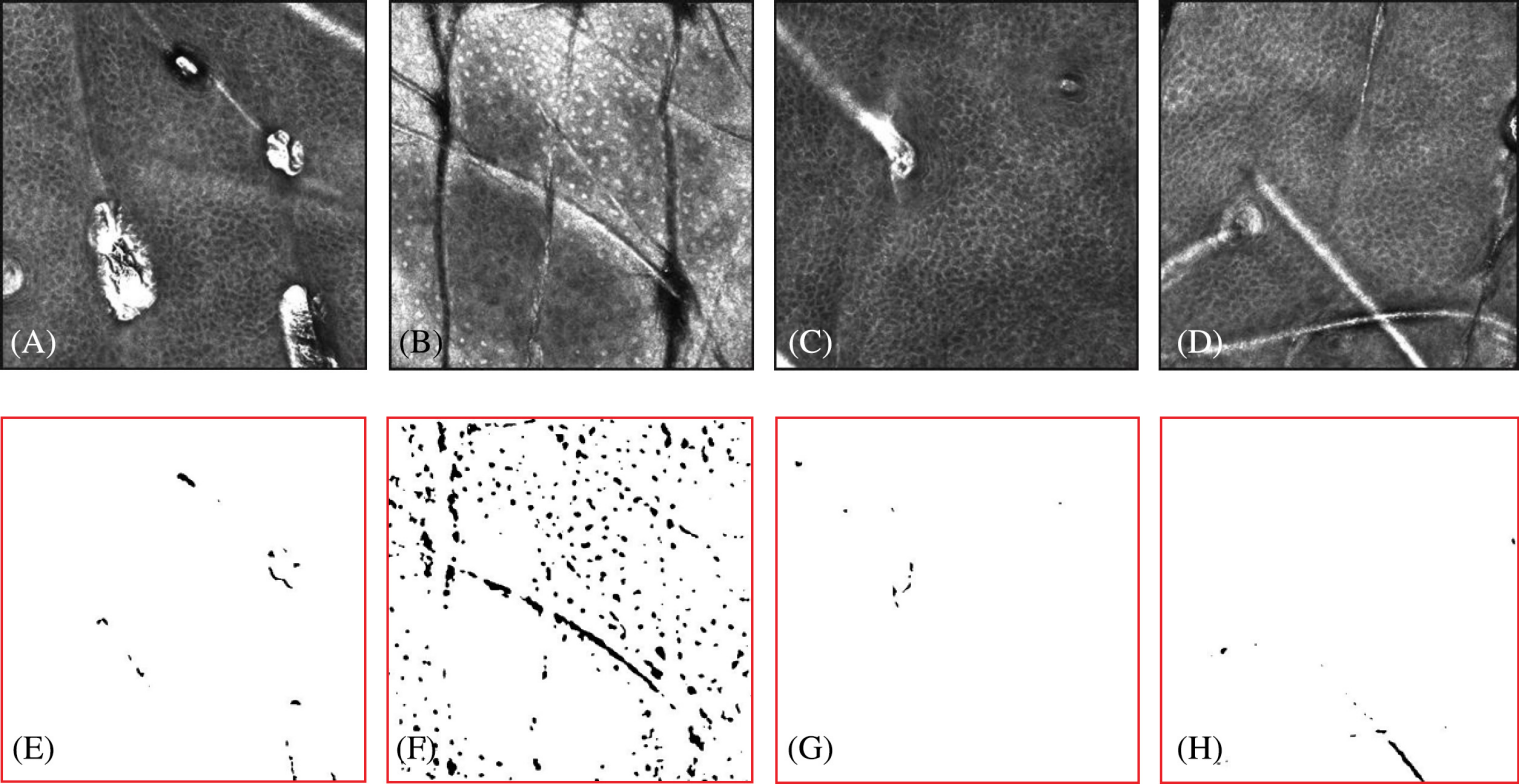
Epidermis (subject 1) Representative original Vivascope images (top row, A–D) and the corresponding processed images (bottom row, E–H) to quantify inflammatory cells in the epidermis. (A) Pre‐peel, (B) 5 min post‐peel, (C) 48 h post‐peel, and (D) 9 days post‐peel

The original Vivascope images were processed, background corrected or filtered (Figure 3) to quantify inflammatory cells (Chart 2) based on their circular shape and high intensity contrast. In subjects 1 and 2, inflammatory cells were accurately discriminated from background as they had uniform punctuated appearance. However, inflammatory cells in subjects 3 and 4 had some chances of error, as it was hard to accurately discriminate inflammatory cells from melanin‐containing cells and the fluid, which all gave similar high intensity contrast and sponge‐like appearance.

#### 3.1.2 Dermal epidermal junction and melanin

**FIGURE 4:**
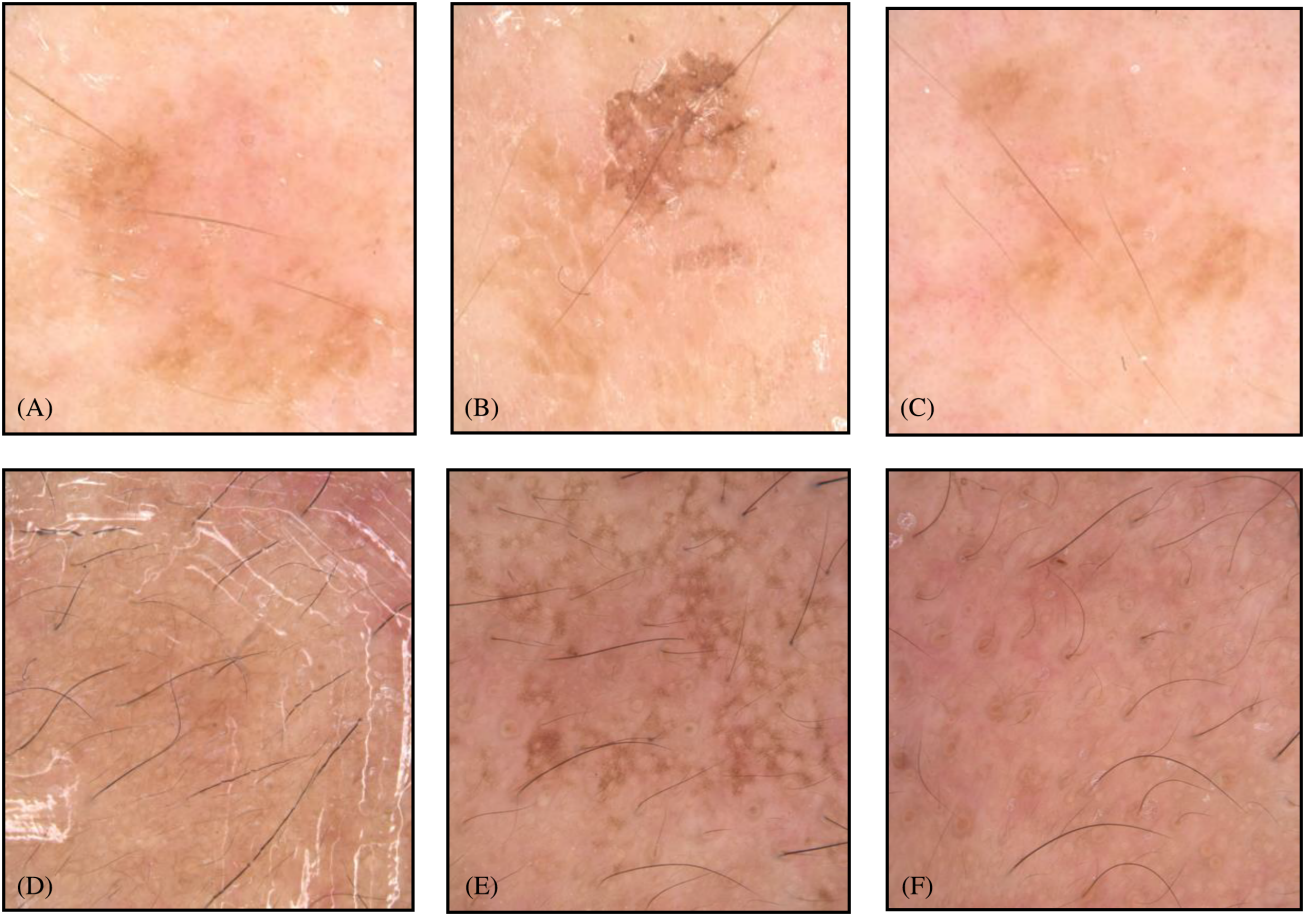
Dermoscopic images of subject 3 (top row A, B, and C) and subject 4 (bottom row D, E, and F) showing changes in skin tone (surface changes) over the course of chemical peeling. (A) Pre‐peel, (B) 48 h post‐peel, (C) 9 days post‐peel. Melanin lifting due to transfer if melanin from basal layer to suprabasal layer is clearly evident at 48 hours post peel (B and E) in both the subjects

RCM is an effective tool in visualizing the dermal‐epidermal junction (DEJ) in darker skin individuals (subject 4) or lighter skin types but prominent dark patches (subject 3). A clear change in skin tone or dark patches was observed under the dermoscope (Figure 4), showing improvement in skin tone, and pre‐peel versus day 9. Interestingly, a worsening of skin tone (dyspigmentation) was observed, pre‐peel versus 48 h (Figure 4). When looking at corresponding Vivastack® videos (1‐3 of subject 4), we observed a strong correlation between dermoscope and Vivascope information to conclude that chemical peeling induced melanin redistribution, and improved skin tone or dark patches. Before peeling, the majority of the melanin is localized to basal rings in DEJ, and has limited melanin content in suprabasal epidermal layers. However, relative to pre‐peeling, after 48 h of peeling, the melanin intensity has decreased in basal DEJ, and increased in suprabasal layer. This redistribution of melanin in response to chemical peel could be called melanin migration or lifting and can be correlated with skin's defense action to try to protect cells in superficial epidermis, melanin cap around nucleus of keratinocytes is clearly evident in 48 h video (2). Similar findings on melanin redistribution in response to injury has been reported by another study.^12^ However, the study was conducted using the classical invasive approach (biopsy followed by histochemical staining to image melanin), and tape stripping was used as an injury model to activate skin's defense system.

**VIDEOS 1–3:**
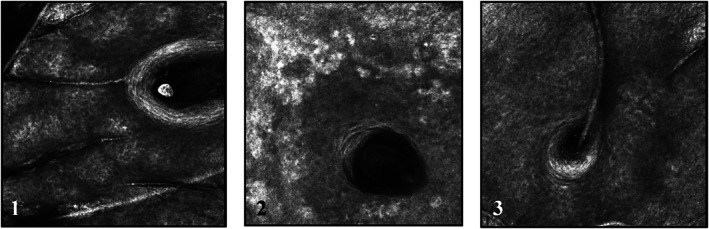
Vivastack® (Vertical or Z‐axis mapping of subject‐4). These video sequences reveal subsurface anatomical changes at pre‐peel (video 1), 48 hours post peel (video 2), and 9 days post peel (video 3).

**FIGURE 5:**
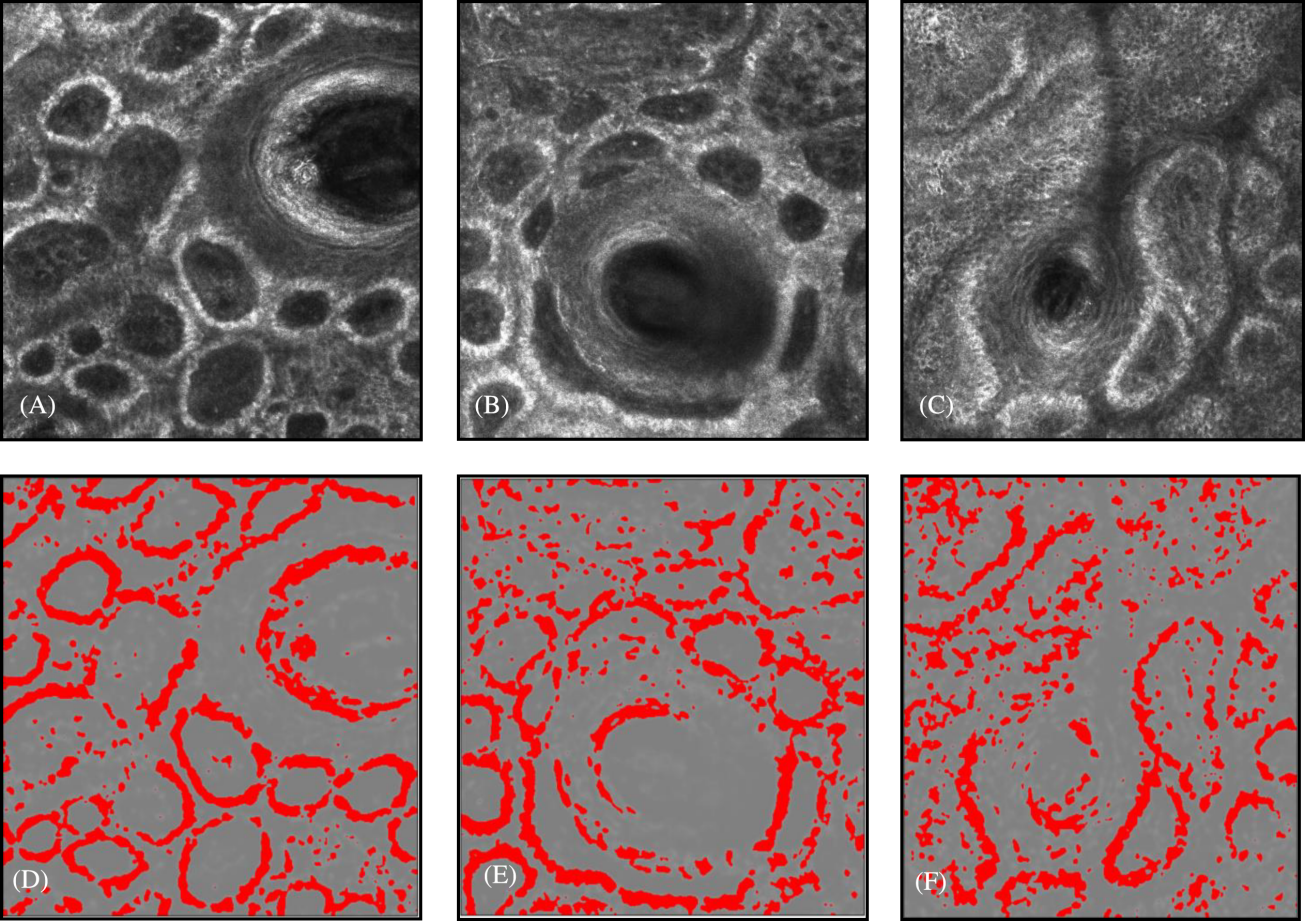
Dermal epidermal junction (subject 4). Representative original Vivascope images (top row, A–C) and the subsequent processed images (bottom row, D–F) to quantify melanin intensity in stratum basalis. (A) Pre‐peel, (B) 48 h post‐peel, and (C) 9 days post‐peel

**CHART 3:**
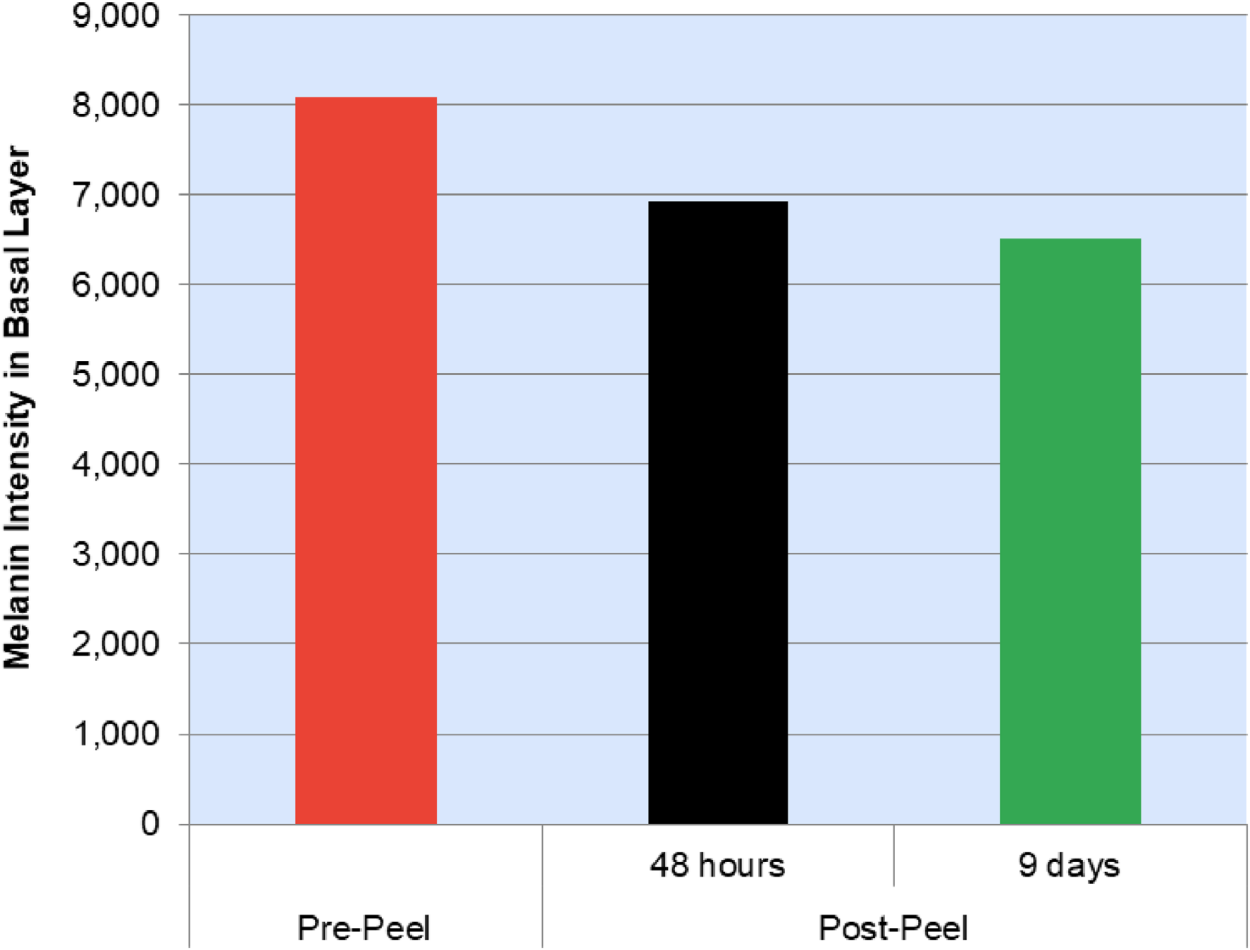
Changes in average melanin intensity in stratum basalis (subject 4)

A significant change in melanin intensity and its organization around the rings in DEJ further supports chemical peel's mode of action on melanin (Figure 5 and Chart 3). DEJ rings became less prominent than before on the 9th day and melanin distribution became even patchier. Patchy distribution of melanin can be visualized in original images and clearly appreciated in processed images (Figure 5). The average melanin intensity at the stratum basalis progressively decreased from pre‐peel to post‐peel (Chart 3).

#### 3.1.3 Superficial dermis

**FIGURE 6:**
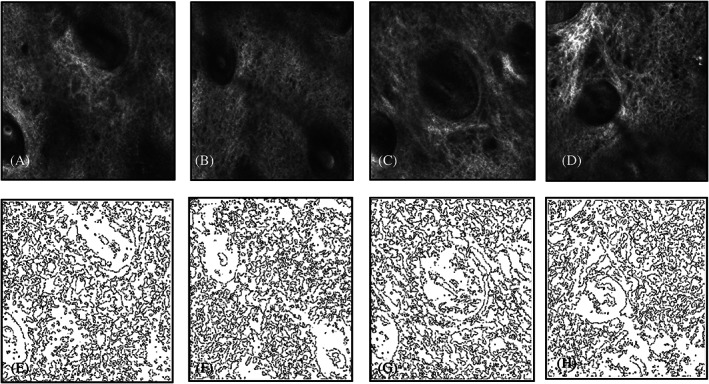
Papillary dermis (subject 1). (A) Baseline, (B) 5 mins post‐peel, (C) 48 h post‐peel, (D) 9 days post‐peel processed confocal images of papillary dermis (subject 1), (E) baseline, (F) 5 min post‐peel, (G) 48 h post‐peel, and (H) Nine days post‐peel

**CHART 4:**
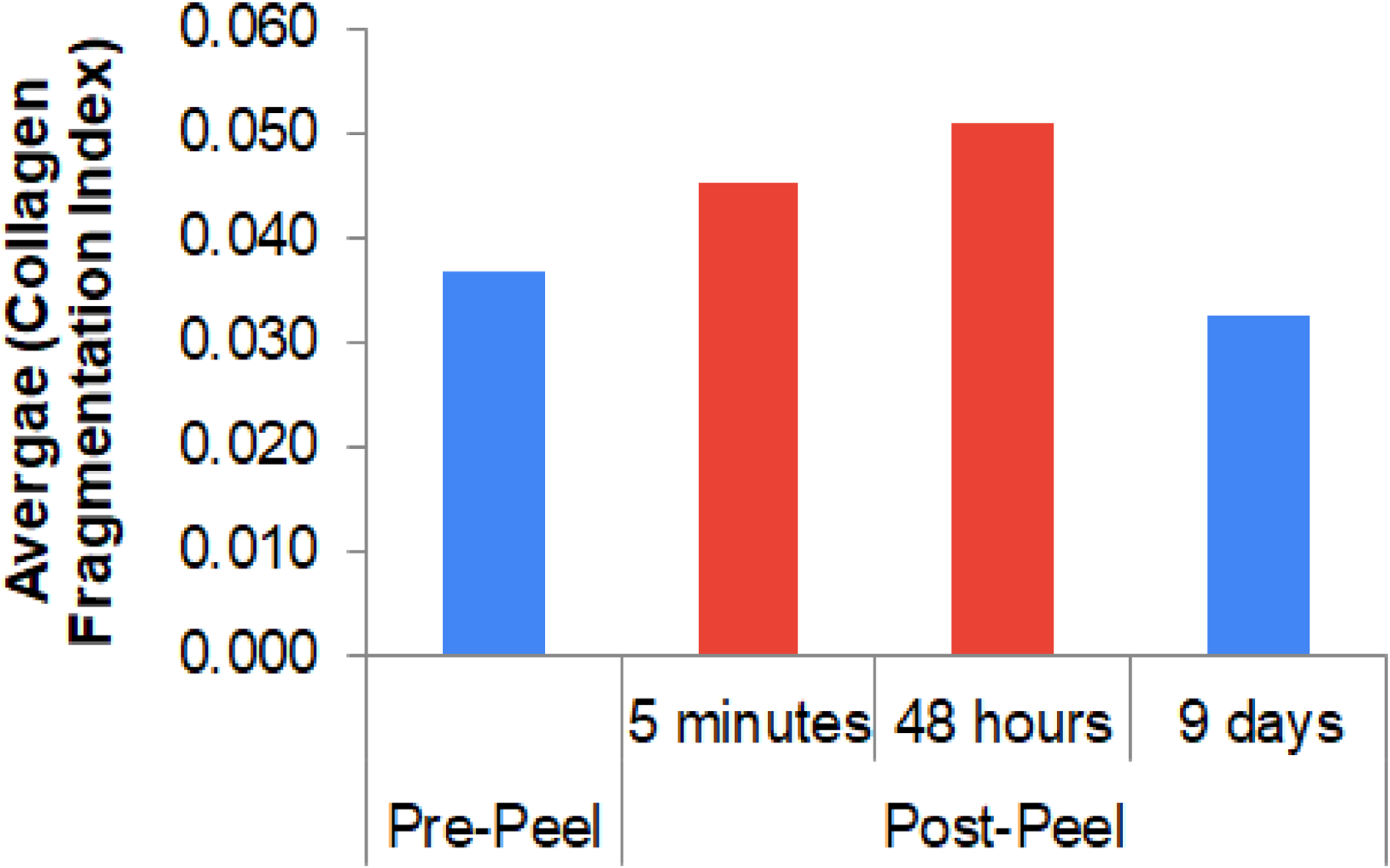
Average collagen fragmentation index (subject 1) (lower the fragmentation index, better the fibrous network)

The superficial papillary dermis can be seen in RCM at an average depth of 110–150 μm.^6^ Collagen in the papillary dermis was analyzed post‐peel to track sequential changes (Figure 6). Fragmentation of collagen fibrous network is instant (5 min), continues for 48 h and then recovers to fibrous network on day 9 (even better than pre‐peel). On the 9th day post‐peel, collagen fibers appear longer and well organized with more parallel alignment. Quantitative analysis of collagen fragmentation index (CFI) in subject 1 reveals that fragmentation was highest at 48 h post‐peel (0.050). CFI was lowest at the 9th day post‐peel (0.032), which was even lower than the pre‐peel level (Chart 4). Similar collagen remodeling profile was observed in subject 4 (data not shown). Increase in collagen and other components of dermal extracellular matrix like elastin and glycosaminoglycans have been reported.^7^, ^9^

#### 3.1.4 Changes visible to the human eye

**FIGURE 7:**
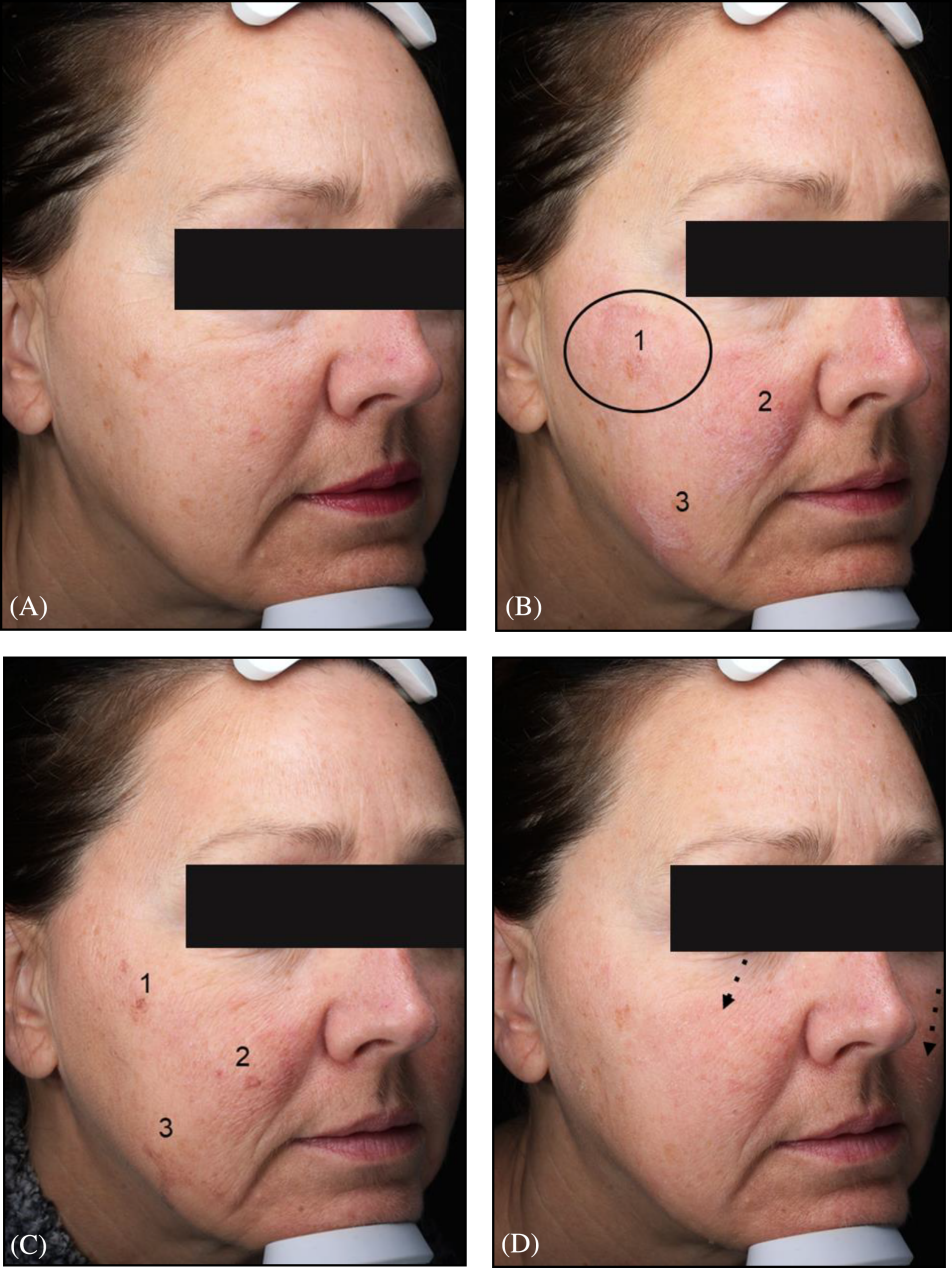
Clinical pictures taken by Visia (subject 3, single peel but two layers). (A) Pre‐peel, (B) 5 min post‐peel, (C) 48 h post‐peel, (D) and 9 days post‐peel. Circle represents the region of interest imaged by Vivascope to monitor anatomical changes after peeling, a rectangular stamp “optical window” of the Vivascope on skin is clearly observed. Numbers 1, 2, and 3 represent three areas of frosting (5 min) and the corresponding hyperpigmentation “melanin migration/lifting” (48 h). Dotted arrows highlight some sign of exfoliation on cheeks on day 9

Visible signs of chemical peeling like frosting and exfoliation were evident in some but not all subjects. Subjects had a more luminescent face on day 9 post‐peel as compared to pre‐peel. Improvement in skin tone/pigmentation was also visibly appreciated by both, patient as well as physician. Some temporary redness (highest at 5 min) was observed in subjects, which corroborates with our Vivascope findings on inflammation. Although some visual redness on day 9 was also observed in some subjects, there was no evidence of inflammatory cells on day 9 under Vivascope (Chart 2). We did not find any direct correlation between the number of peel layers applied with degree or trend of anatomical changes. Further there was no direct correlation between degree of frosting/exfoliation and efficacy. For example, subject 3 (Figure 7) showed the highest level of frosting among all subjects (applied two layers of peel). Except increase in pigmentation at three frosted areas (indicates migration/lifting of melanin at 48 h as sign of skin's defense system activation), no other correlation could be made.

## 4 DISCUSSION

Chemical peeling is a very popular procedure, however, not much is known about its mechanism of action. In our case studies, we used an FDA‐approved non‐invasive diagnostic medical device (Vivascope 1500) to elucidate the mysterious mechanism of chemical peeling.

We were able to visualize (Vivascope) as well as quantify (ConfoScan) the changes in markers or indicators of skin injury and healing. We found clear evidence of instant inflammation (within 5 min of chemical peel application) in all four subjects recruited for this study. The inflammation was temporary with no significant number of inflammatory cells on day 9 after peeling. In subjects with high melanin content (subject 4) or dark patches (subject 3), signs of melanin redistribution were observed, melanin redistribution was clearly evident at 48 h in both the subjects. These findings on melanin redistribution at 48 h post‐peel are in agreement with previous study that used tape stripping as the injury model.^12^ Collagen remodeling was noticed in two subjects with lighter skin tone, showing a more fibrous network of long collagen fibers on day 9 post‐peel versus pre‐peel. The increase in fibrous network of collagen after chemical peeling corroborates with previous studies that analyzed punch biopsies after chemical peeling.^7^, ^9^ On the basis of depth of injury by the chemical peel, melanin redistribution in DEJ and collagen remodeling in papillary dermis,^1^ and our previous findings on synergistic action of two exfoliating agents in the peel (TCA and lactic acid),^4^ we can infer that the professional peel used in this study performed like a medium depth peel but at lower concentration of acids (10% TCA and 20% lactic acid). Similar findings have been reported by another study, they found 15% TCA peel blended with hydroxy acid (combination peeling) to deliver better efficacy and tolerance than classical medium depth peel using a single TCA ingredient at 35% concentration.^13^ The aforementioned structural (collagen, melanin) and functional changes (inflammation) in the skin are the hallmarks of the efficacy of chemical peels in improving skin tone (dyspigmentation) and firmness, accompanied by temporary redness.

Indeed exfoliation and acidic pH to achieve exfoliation (remove dead and slow growing cells to increase cell turnover of healthy cells) is the hallmark of chemical peeling. However, based on observations we suggest, patients and physicians should consider using progressive mild superficial peeling approach over aggressive approach of using strong concentrated acids if the indication of use is to correct photodamage or any other skin condition that requires correction of dyspigmented or wrinkles skin. A carefully tailored superficial and progressive peeling approach can deliver the same or even better efficacy and with much less downtime as compared to a strong aggressive monopeeling approach. Although peeling is usually supplemented with post‐peel care or maintenance products like skin brighteners^8^ and poly hydroxy acids,^10^ peeling alone has the capability to reduce pigmentation and signs of aging.

## 5 CONCLUSIONS

Through non‐invasive optical biopsy using Vivascope and image processing using ConfoScan, we were able to clearly visualize and quantify structural and functional changes in markers of chemical peel's action on skin, to conclude that the chemical peel: (1) induced temporary chemical injury (inflammation) that triggers skin's defense system (melanin redistribution) to initiate healing (collagen remodeling), (2) performed like a classical medium depth peel.

## AUTHOR CONTRIBUTIONS

Shazli Razi contributed to conception and design, data collection, data interpretation and manuscript production. Vinay Bhardwaj contributed to data interpretation and manuscript production. Samantha Ouellette contributed to image acquisition/data collection, analyses confocal interpretation and editing. Samavia Khan contributed to image acquisition/data analyses manuscript editing, and proof reading. Chloe Azadegan contributed to data analysis with Confoscan software. Thomas Boyd contributed intellectually to manuscript production. Babar Rao contributed to conception and design of the work, and final review of manuscript.

## FUNDING INFORMATION

This research work was funded by Colgate‐Palmolive.

## CONFLICT OF INTEREST

Babar Rao is a consultant for Caliber ID (The manufacturer of VivaScope®). The other authors have no conflict of interest to disclose.

## ETHICS STATEMENT

The study protocol was reviewed and approved by Advarra IRB.

## Supporting information


**Videos 1‐3.** Vivastack® (Vertical or Z‐axis mapping of subject‐4). These video sequences reveal subsurface anatomical changes at pre‐peel (video 1), 48 hours post peel (video 2), and 9 days post peel (video 3).

## Data Availability

All data generated or analyzed during this study are included in this article. Further enquiries can be directed to the corresponding author.

## References

[1] Soleymani T , Lanoue J , Rahman Z . A practical approach to chemical peels: a review of fundamentals and step‐by‐step algorithmic protocol for treatment. J Clin Aesthet Dermatol. 2018;11(8):21‐28. PMC6122508 30214663

[2] O'Connor AA , Lowe PM , Shumack S , Lim AC . Chemical peels: a review of current practice. Australas J Dermatol. 2018;59:171‐181. 29064096 10.1111/ajd.12715

[3] Briden ME . Alpha‐hydroxyacid chemical peeling agents: case studies and rationale for safe and effective use. Cutis. 2004;73(2 Suppl):18‐24. 15002658

[4] Bhardwaj V , Sharma K , Maksimovic S , Fan A , Adams‐Woodford A , Mao J . Professional‐grade TCA‐lactic acid chemical peel: elucidating mode of action to treat photoaging and hyperpigmentation. Front Med. 2021;8:1‐9. doi:10.3389/fmed.2021.617068 PMC7928281 33681250

[5] Tang SC , Yang JH . Dual effects of alpha‐hydroxy acids on the skin. Molecules. 2018;23(4):863. doi:10.3390/molecules23040863 29642579 PMC6017965

[6] Yamamoto Y , Uede K , Yonei N , Kishioka A , Ohtani T , Furukawa F . Effects of alpha‐hydroxy acids on the human skin of Japanese subjects: the rationale for chemical peeling. J Dermatol. 2006;33(1):16‐22. doi:10.1111/j.1346-8138.2006.00003.x 16469079

[7] Khater MH . A comparative study of glycolic acid versus mixture of Trichloroacetic acid and glycolic acid peeling for treatment of three benign epidermal lesions. J Clin Exp Dermatol Res. 2014;5:1‐7.

[8] Goberdhan LT , Mehta RC , Aguilar C , Makino ET , Colvan L . Assessment of a superficial chemical peel combined with a multimodal, hydroquinone‐free skin brightener using in vivo reflectance confocal microscopy. J Drugs Dermatol. 2013;12(3):S38‐S41. 23545932

[9] Butler PE , Gonzalez S , Randolph MA , Kim J , Kollias N , Yaremchuk MJ . Quantitative and qualitative effects of chemical peeling on photo‐aged skin: an experimental study. Plast Reconstr Surg. 2001;107(1):222‐228. doi:10.1097/00006534-200101000-00036 11176627

[10] Sadick N , Edison BL , John G , Bohnert KL , Green B . An advanced, physician‐strength retinol Peel improves signs of aging and acne across a range of skin types including Melasma and skin of color. J Drugs Dermatol. 2019;18(9):918‐923. 31524348

[11] Salvador G . Reflectance Confocal Microscopy of Cutaneous Tumors. CRC Press, Taylor & Francis Group; 2017.

[12] Joly‐Tonetti N , Wibawa J , Bell M , Tobin D . An explanation for the mysterious distribution of melanin in human skin ‐ a rare example of asymmetric (melanin) organelle distribution during mitosis of basal layer progenitor keratinocytes. Br J Dermatol. 2018;179:1115‐1126. doi:10.1111/bjd.16926 29956303

[13] Kubiak M , Mucha P , Rotsztejn H . Comparative study of 15% trichloroacetic acid peel combined with 70% glycolic acid peel for the treatment of photodamaged facial skin in aging women. J Cosmet Dermatol. 2020;19(1):137‐146. doi:10.1111/jocd.13171 31603267

